# Aberration correction expands the treatment envelope for histotripsy in the liver

**DOI:** 10.1088/1361-6560/ae222c

**Published:** 2025-12-05

**Authors:** Ellen Yeats, Ning Lu, Jonathan Sukovich, Zhen Xu, Timothy L Hall

**Affiliations:** 1 Department of Biomedical Engineering, University of Michigan, Ann Arbor, MI, United States of America; 2 Department of Radiology, Stanford University, Palo Alto, CA, United States of America; 3 Department of Radiology, University of Michigan, Ann Arbor, MI, United States of America; 4 Department of Neurosurgery, University of Michigan, Ann Arbor, MI, United States of America

**Keywords:** therapeutic ultrasound, histotripsy, phase aberration, soft tissue, simulation

## Abstract

*Objective.* Histotripsy is a non-invasive, non-thermal, and non-ionizing tissue ablation method based on high-amplitude pulses of focused ultrasound that has been approved by the federal Food and Drug Administration for the treatment of liver tumors. However, histotripsy currently cannot treat all locations in the liver due to attenuation of the pressure amplitude at the focus by sound-blocking ribs and air pockets and by phase aberration, or de-focusing of the ultrasound pulses by heterogeneous bodily tissues. Previous work suggests that correcting for phase aberration could increase the focal pressure to expand the treatment envelope (the treatable region) for histotripsy in the liver. The objective of this study was to investigate the effect of aberration correction on the treatment envelope. *Approach*. Acoustic propagation was simulated in the human body using a linear model (k-Wave) for a histotripsy phased array of similar dimensions to the current Histosonics Edison® clinical device and anatomical data from 10 subjects of varying body size. *Main results*. We find that aberration correction increases the focal pressure throughout the liver to substantially expand the treatment envelope (from 67% to 81% of the liver volume, on average, by linear estimations). *Significance*. The study suggests that aberration correction could help enable non-invasive, non-thermal histotripsy treatments for a broader patient population.

## 1. Introduction

Histotripsy is a non-invasive, non-thermal, and non-ionizing tissue ablation method that was recently approved by the United States Food and Drug Administration (FDA) for the treatment of liver tumors (Xu *et al* 2021, Wehrle *et al* 2025). Histotripsy focuses short, high amplitude ultrasound pulses into the body to generate acoustic cavitation bubbles that energetically expand and collapse, exerting localized high strain rate forces to mechanically fracture and ultimately liquefy the target tissue (tumor) while sparing surrounding healthy tissues (Xu *et al* 2008, Khokhlova *et al* 2015). By selectively destroying tumors without incisions, heat, or ionizing radiation, histotripsy avoids comorbidities associated with other tumor removal methods like surgery, thermal ablation, and radiation beam therapy (Smolock *et al* 2022, Knott *et al* 2023). Furthermore, in addition to demonstrating the efficacy and safety of histotripsy ablation (Mendiratta-Lala *et al* 2024, Sandilos *et al* 2024, Ziemlewicz *et al* 2025), clinical data have suggested that histotripsy can help potentiate an anti-tumor immune response (Vidal-Jové *et al* 2021). Consequently, histotripsy has become an attractive option for treating liver cancer.

However, histotripsy currently cannot be used at all locations in the liver (Wah *et al* 2023). To generate cavitation for histotripsy ablation, the ultrasound pulses must focus at the target with sufficient amplitude to produce a large rarefactional pressure amplitude (⩾26 MPa in the liver for intrinsic threshold histotripsy) (Maxwell *et al* 2013, Lin *et al* 2014). For deep and/or cranial portions of the liver, intervening ribs, lung, and bowel obstruct ultrasound, restricting the acoustic window (region of unobstructed path to the focus) and limiting the pressure amplitude (Hall *et al* 2010, de Greef *et al* 2015, Knott *et al* 2021, Tsang *et al* 2021, Kisting *et al* 2024). In addition, intervening heterogeneous soft tissues cause the speed of sound propagation to vary along the ultrasound path, producing phase errors that undermine constructive interference at the focus and further decrease the pressure amplitude (Fan and Hynynen 1992, Macoskey *et al* 2018, Bobina *et al* 2021, Magnier *et al* 2025). This effect, called phase aberration, decreases the pressure amplitude at the histotripsy focus in caudal regions of the human liver by ∼50%, on average (Yeats *et al* 2022). In some cases, loss of focal pressure can be compensated by increasing the acoustic power output from the histotripsy transducer (Gao *et al* 2014, Khokhlova *et al* 2019). However, increased acoustic power elevates the risk of unsafe heating, especially if strongly absorbing tissues like ribs are in the path (Kim *et al* 2014). Therefore, by critically limiting the focal pressure, phase aberration and acoustic obstructions like ribs restrict the treatment envelope (range of locations where histotripsy is feasible), ultimately reducing the number of patients who can receive histotripsy treatment.

Recently, researchers have developed devices and methods adapting earlier work from ultrasound imaging (Flax and O’Donnell 1988, Nock *et al* 1989, Wu *et al* 1992, Ng *et al* 1994) and thermal focused ultrasound (Gateau *et al* 2010, Jing *et al* 2012, Mougenot *et al* 2012, Leung *et al* 2021) to correct phase aberration during histotripsy. New multi-element histotripsy transducers (phased arrays) can correct for aberration by varying the per-element transmit phase across the array aperture to compensate for the phase errors at the focus (Hynynen and Jones 2016, Bawiec *et al* 2021, Stocker *et al* 2022). These phase errors are generally not known *a priori* but can be determined either by numerically simulating ultrasound propagation through the body with pre-treatment medical images (White *et al* 2008, Jones and Hynynen 2015, Dillon *et al* 2018, Bancel *et al* 2021, Rosnitskiy *et al* 2024, Manuel *et al* 2025) or, more recently, by using a phased array with receive-capable transducer elements to sense acoustic signals from the body and directly measure phase differences (Thomas *et al* 2021, Yeats *et al* 2023). *In vitro* experiments with receive-capable histotripsy phased arrays have shown that correction using acoustic signals can restore nearly all focal pressure amplitude lost to phase aberration from abdominal soft tissues (Thomas *et al* 2021, Yeats *et al* 2023). Furthermore, aberration correction with receive-capable phased arrays substantially reduced the acoustic input power required to perform histotripsy in porcine liver *in vivo* (Thomas *et al* 2022, Yeats *et al* 2024). These results suggest that aberration correction could substantially increase the focal pressure and/or reduce the input power needed to achieve focal pressures sufficient for cavitation to improve safety and expand the treatment envelope of histotripsy in the human liver. However, the devices and algorithms for aberration correction increase the complexity of histotripsy treatments (Yeats and Hall 2023, Lu *et al* 2024). Therefore, before implementing aberration correction, it will be useful to know how much aberration correction will improve focusing and the treatment envelope for histotripsy in the liver.

The objective of this study was to assess the effect of aberration correction on the treatment envelope of histotripsy in the human liver. Our approach was to computationally simulate focusing a histotripsy beam throughout the liver both with and without aberration correction using a linear numerical acoustic propagation model previously validated for investigating soft tissue phase aberration in intrinsic threshold histotripsy (Yeats *et al* 2022). For clinical relevance, we simulate a phased array with similar dimensions to the current FDA-approved Histosonics Edison® transducer and use anatomical data from 10 adult subjects of varying body composition and gender.

## 2. Methods

### 2.1. Histotripsy phased array geometry

**Figure 1.**
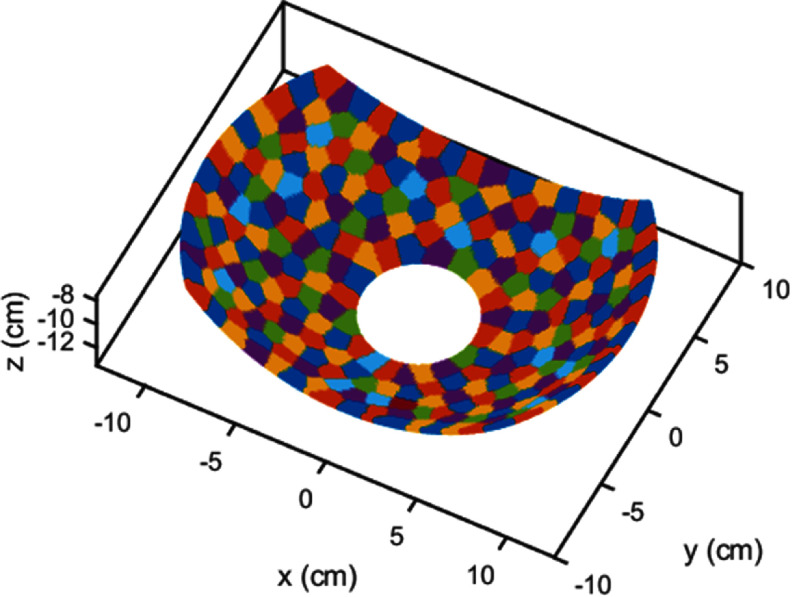
The simulated histotripsy transducer with 256 equal-area polygon shaped elements. Elements are shown in different colors for visual contrast.

The treatment envelope was mapped for the geometry of a custom-built histotripsy transducer from our lab (Stocker *et al* 2022) which has similar dimensions to the Histosonics® Edison® device currently in clinical use for histotripsy ablation of liver tumors. The modeled transducer had a truncated spherical aperture with maximum lateral extents of 23 and 15.7 cm, a 14.2 cm focal radius, and a 4 cm circular cutout at the apex. The central frequency ${f_{\text{c}}}$ was 750 kHz. To simulate a phased array, the transducer aperture was sub-divided into 256 equal-area elements with irregular polygon shapes arranged pseudo-randomly following the method described by Rosnitskiy *et al* (2018) which has been used to design and fabricate therapeutic arrays previously (Tsysar *et al* 2024, Rosnitskiy *et al* 2025). The geometry of the simulated phased array is shown in figure 1.

### 2.2. Anatomical data

**Figure 2.**
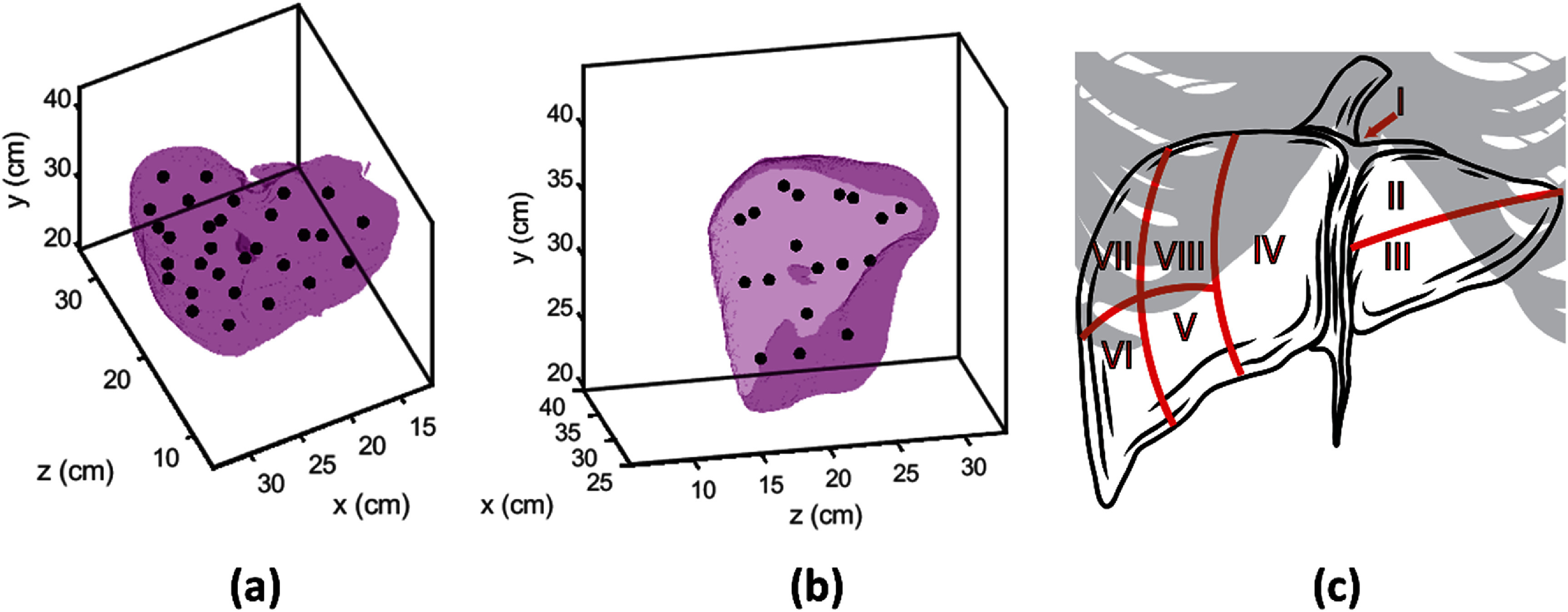
(a) Purple volume showing the segmented liver for one anatomical subject. Black dots denote 30 target locations dispersed throughout liver. (b) Sagittal section of the segmented liver volume. (c) Couinaud segments of the liver. Couinaud segment I is posterior to the other segments and anterior to the inferior vena cava. Ribs (gray) obstruct lateral, cranial portions of the liver.

The acoustic path through the human abdomen was modeled using 3D CT scans (slice spacing: 1 mm, pixel size: 0.68–0.94 mm) of 10 de-identified adult subjects obtained from an open access medical imaging repository (Clark *et al* 2013, Roth *et al* 2014, Seff *et al* 2014, 2015). The subjects included 5 males and 5 females. The CT images were segmented into air (lung, bowel), bone, cartilage, skin, fat, and other soft tissue using Hounsfield unit thresholds as described previously (Yeats *et al* 2022). The liver was segmented manually and then sub-divided into 30 equal sub-volumes (average over subjects: 75 cm^3^, range: 40–125 cm^3^). The center of mass of each sub-volume was set as a target for acoustic focusing (see figures 2(a) and (b)). To aid clinical reference, the liver of each subject was also manually divided into Couinaud segments, a set of 8 anatomical regions often used to describe liver tumor location (Couinaud 2000). An anatomical illustration of the Couinaud segments is given in figure 2(c). The median number of focusing targets per segment and average segment volumes are given in table 1.

**Table 1.** Size and aperture blockage of liver Couinaud segments. The average volume, number of focusing targets, and percent blockage of the transducer aperture are provided for each Couinaud segment of the liver.

Couinaud segment	Volume	Number of targets in segment	Percent aperture blocked
	Mean ± standard deviation	Median (minimum, maximum)	Mean ± standard deviation
I	57 ± 36 cm^3^	1 (0, 1)	47 ± 13%
II	315 ± 54 cm^3^	4 (2, 8)	21 ± 21%
III	150 ± 62 cm^3^	2 (1, 6)	27 ± 20%
IV	484 ± 299 cm^3^	6 (4, 10)	33 ± 26%
V	336 ± 70 cm^3^	5 (3, 7)	22 ± 17%
VI	143 ± 100 cm^3^	2 (0, 5)	27 ± 13%
VII	303 ± 146 cm^3^	4 (2, 8)	52 ± 14%
VIII	376 ± 204 cm^3^	5 (3, 12)	50 ± 15%

### 2.3. Acoustic window maximization

To map the treatment envelope, we must estimate the maximum possible focal pressure for each target location in the liver. The focal pressure is limited both by aberration and obstructions like ribs and air pockets. For a given focusing target, the amount of obstruction in the acoustic path depends on the orientation of the transducer with respect to the body. For example, obstruction by ribs can be reduced when targeting subcostal portions of the liver by tilting the cranial-most portion of the transducer away from the abdomen. Thus, for each focusing target, obstruction was minimized by finding the transducer orientation that maximized the acoustic window, or the free (unblocked) area of the transducer aperture.

**Figure 3.**
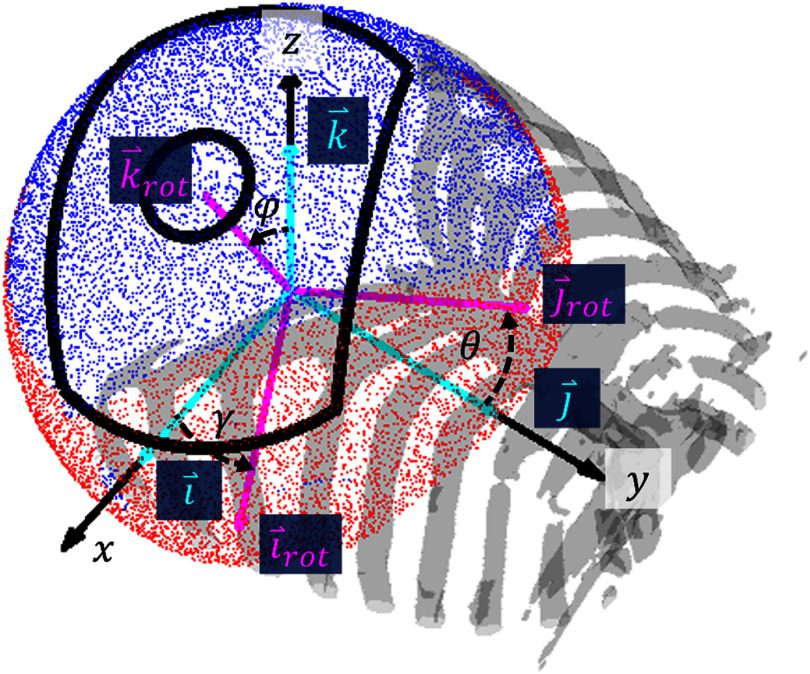
To calculate the acoustic window, rays (ray endpoints shown as red and dark blue dots) were projected through anatomic image space from the target (origin) to a sphere with focal length (14.2 cm) radius. The transducer outline (thick black line) was numerically rotated around the sphere by rotating axis unit vectors $\overset{{\scriptscriptstyle\rightharpoonup}} {{i}} $, $\overset{{\scriptscriptstyle\rightharpoonup}} {{j}} $, and $\overset{{\scriptscriptstyle\rightharpoonup}} {{k}} $ (light blue) by angles $\theta $, $\varphi $, and $\gamma $ (black dashed arrows) about the *x, y,* and *z* axes (black solid arrows) respectively to a rotated reference frame with axis unit vectors ${\overset{{\scriptscriptstyle\rightharpoonup}} {{i}}\!\!_{{\text{rot}}}}$, ${\overset{{\scriptscriptstyle\rightharpoonup}} {{j}}\!\!_{{\text{rot}}}}$, and ${\overset{{\scriptscriptstyle\rightharpoonup}} {{k}}\!\!_{{\text{rot}}}}$ (pink). At each orientation, the number of blocked (red) and unblocked (dark blue) rays in the transducer outline were counted to compute the acoustic window.

For each target, a range of possible transducer positions was defined by projecting straight-line rays from the target out of the body in anatomical image space to a hemispherical surface with radius equal to the focal length of the transducer (14.2 cm). The maximum distance between rays on the surface was 1.3 mm (0.65 ${\lambda _{\text{c}}}$, where ${\lambda _{\text{c}}}$ = 2 mm is the wavelength in water at 750 kHz). Rays that intersected voxels with air or rib were marked as ‘blocked.’ Then, the acoustic window was calculated for different rotations of the transducer as defined by the angles of the transducer axes ($\theta $, $\varphi $, and $\gamma $) with the $x$, $y$ and $z$ axes of the anatomical image, which run right-to-left, cranial-to-caudal, and ventral-to-dorsal along the body, respectively (see figure 3). The angles $\theta $, $\varphi $, and $\gamma $ were varied from −70 to 70°, −70 to 70°, and −45–45°, respectively, in 5° increments. Positions where the transducer intersected skin were excluded. For each combination of angles, transducer rotations were numerically simulated by first rotating the $x$, $y$, and $z$ axis unit vectors $\overset{{\scriptscriptstyle\rightharpoonup}} {\boldsymbol{i}} $, $\overset{{\scriptscriptstyle\rightharpoonup}} {\boldsymbol{j}} $, and $\overset{{\scriptscriptstyle\rightharpoonup}} {\boldsymbol{k}} $ by $\theta $, $\varphi $, and $\gamma $ using

\begin{equation*}\begin{array}{*{20}{c}} {{{\overset{{\scriptscriptstyle\rightharpoonup}} {\boldsymbol{i}} }_{{\boldsymbol{rot}}}} = \cos \varphi \cos \gamma { }\overset{{\scriptscriptstyle\rightharpoonup}} {\boldsymbol{i}} + \sin \gamma \overset{{\scriptscriptstyle\rightharpoonup}} {\boldsymbol{j}} - \sin \varphi \cos \gamma \overset{{\scriptscriptstyle\rightharpoonup}} {\boldsymbol{k}} } \end{array}\end{equation*}

\begin{equation*}\begin{array}{*{20}{c}} {{{\overset{{\scriptscriptstyle\rightharpoonup}} {\boldsymbol{j}} }_{{\boldsymbol{rot}}}} = - \sin \gamma \overset{{\scriptscriptstyle\rightharpoonup}} {\boldsymbol{i}} + \cos \theta \cos \gamma \overset{{\scriptscriptstyle\rightharpoonup}} {\boldsymbol{j}} + \sin \theta \cos \gamma \overset{{\scriptscriptstyle\rightharpoonup}} {\boldsymbol{k}} } \end{array}\end{equation*}

\begin{equation*}\begin{array}{*{20}{c}} {{{\overset{{\scriptscriptstyle\rightharpoonup}} {\boldsymbol{K}} }_{{\boldsymbol{rot}}}} = - \sin \varphi \overset{{\scriptscriptstyle\rightharpoonup}} {\boldsymbol{i}} + \sin \theta \cos \varphi \overset{{\scriptscriptstyle\rightharpoonup}} {\boldsymbol{j}} + \cos \theta \cos \varphi \overset{{\scriptscriptstyle\rightharpoonup}} {\boldsymbol{k}} } \end{array}\end{equation*}

where ${\overset{{\scriptscriptstyle\rightharpoonup}} {\boldsymbol{i}} _{{\boldsymbol{rot}}}}$, ${\overset{{\scriptscriptstyle\rightharpoonup}} {\boldsymbol{j}} _{{\boldsymbol{rot}}}}$, and ${\overset{{\scriptscriptstyle\rightharpoonup}} {\boldsymbol{k}} _{{\boldsymbol{rot}}}}$ are the axis unit vectors of the rotated reference frame. Then, the coordinates ${x_r},\,{y_r},$ and ${z_r}$ of each ray $\overset{{\scriptscriptstyle\rightharpoonup}} {\boldsymbol{r}} $ were projected onto the rotated reference frame using

\begin{equation*}\begin{array}{*{20}{c}} {{x_{r,\,{\text{proj}}}} = {{\overset{{\scriptscriptstyle\rightharpoonup}} {\boldsymbol{i}} }_{{\boldsymbol{rot}}}} \cdot \overset{{\scriptscriptstyle\rightharpoonup}} {\boldsymbol{r}} } \end{array}\end{equation*}

\begin{equation*}\begin{array}{*{20}{c}} {{y_{r,\,{\text{proj}}}} = {{\overset{{\scriptscriptstyle\rightharpoonup}} {\boldsymbol{j}} }_{{\boldsymbol{rot}}}} \cdot \overset{{\scriptscriptstyle\rightharpoonup}} {\boldsymbol{r}} } \end{array}\end{equation*}

\begin{equation*}\begin{array}{*{20}{c}} {{z_{r,\,{\text{proj}}}} = {{\overset{{\scriptscriptstyle\rightharpoonup}} {\boldsymbol{k}} }_{{\boldsymbol{rot}}}} \cdot \overset{{\scriptscriptstyle\rightharpoonup}} {\boldsymbol{r}} } \end{array}\end{equation*}

where $ \cdot $ is the dot product, and ${x_{r,\,{\text{proj}}}}$, ${y_{r,\,{\text{proj}}}}$, and ${z_{r,\,{\text{proj}}}}$ are the coordinates of the ray $\overset{{\scriptscriptstyle\rightharpoonup}} {\boldsymbol{r}} $ in the rotated reference frame. Then, the outline of the transducer aperture was drawn in the rotated coordinate space such that the long transverse, short transverse, and depth axes of the transducer aligned with ${\overset{{\scriptscriptstyle\rightharpoonup}} {\boldsymbol{i}} _{{\boldsymbol{rot}}}}$, ${\overset{{\scriptscriptstyle\rightharpoonup}} {\boldsymbol{j}} _{{\boldsymbol{rot}}}}$, and ${\boldsymbol{\overset{{\scriptscriptstyle\rightharpoonup}} {\boldsymbol{k}}_{{\boldsymbol{rot}}}}}$, respectively. All projected rays lying within the bounds of the transducer aperture were then counted as blocked or unblocked to calculate the acoustic window (the total unblocked aperture area). The acoustic window was calculated for all orientations (unique combinations of $\theta $, $\varphi $ and $\gamma $). Then, of the orientations with acoustic windows within 5% of the maximum acoustic window, the orientation with $\min \left( {{\text{sum}}\left( {\left| \theta \right|,\left| \varphi \right|,\left| \gamma \right|} \right)} \right)$ was selected for the given focusing target to discourage large deviations from normal incidence while maintaining a nearly maximal acoustic window. For targets without blockage, the orientation angles were set to 0°. The average percent blocked aperture after optimization is given by Couinaud segment in table 1. A schematic of the acoustic window calculation is shown in figure 3.

For some deep-lying targets (14% of the liver on average across subjects, min: 0%, max: 27%), the transducer could not be placed within the 14.2 cm hemisphere at any orientation without intersecting the body. For these targets, the transducer was retracted from the target by 1 cm increments and rotated around the corresponding enlarged hemisphere until the transducer could be placed without intersecting the skin. Once the transducer was placed sufficiently far from the body, the acoustic window was optimized for the extended focusing distance following the steps described above. Across all targets, the focusing distance was extended by a minimum of 2 cm and maximum of 3 cm (electronic focal steering), where the focal pressure decreases by 35% and 60%, respectively. Targets requiring extensions >3 cm (9% of the liver on average across subjects, min: 0%, max: 20%) were not considered and assumed to be untreatable in this study.

### 2.4. Numerical acoustic propagation model

Once the acoustic window was maximized for each target, acoustic propagation was simulated to assess focusing quality with and without aberration correction. Acoustic propagation was simulated in the linear regime using a numerical model previously validated for investigating phase aberration in histotripsy (Yeats *et al* 2022). Simulations were performed with k-Wave, a MATLAB Toolbox that numerically solves the wave equation for linear propagation of arbitrarily shaped acoustic sources in heterogeneous, absorbing media as three first-order partial differential equations describing the conservation of momentum, the conservation of mass, and the pressure-density relation, respectively (Treeby and Cox 2010):

\begin{equation*}\begin{array}{*{20}{c}} {\frac{{\partial {\mathbf{u}}}}{{\partial t}} = - \frac{1}{{{\rho _0}}}\nabla p} \end{array}\end{equation*}

\begin{align*}\begin{array}{*{20}{c}} {\frac{{\partial \rho }}{{\partial t}} = - {\rho _0}\nabla \cdot {\mathbf{u}} - {\mathbf{u}} \cdot \nabla {\rho _0} + S} \end{array}\end{align*}

\begin{equation*}\begin{array}{*{20}{c}} {{ }p = { }c_0^2\left( {\rho + {\mathbf{d}} \cdot \nabla {\rho _0} + 2{\alpha _0}\rho \left( {c_0^{y - 1}\frac{\partial }{{\partial t}}{{\left( { - {\nabla ^2}} \right)}^{\frac{y}{2} - 1{ }}} - c_0^y\tan \left( {\frac{{\pi y}}{2}} \right){{\left( { - {\nabla ^2}} \right)}^{\frac{{y + 1}}{2} - 1}}} \right)} \right)} \end{array}\end{equation*}

where ${\mathbf{u}}$ is the particle velocity vector, $p\,$ is the acoustic pressure, $\rho $ is acoustic density, ${c_0}$ is the speed of sound, ${\rho _0}$ is the ambient density, $\,{\text{S}}$ is a source term representing the time rate of injection of mass per unit volume, ${\mathbf{d}}$ is the particle displacement vector, ${\alpha _0}$ is the attenuation coefficient, and $y$ is the power law exponent. In this study, the power law exponent $y$ was set to 1.9, which limits errors from dispersion without significantly altering absorption at sub-MHz frequencies (Treeby and Cox 2010). For speed, k-Wave solves the equations in the spatial frequency domain or k-space, which permits numerically efficient approximations of the spatial and temporal derivatives (Treeby *et al* 2012). In all simulations, space and time were discretized to steps of 0.3 mm (${\lambda _{\text{c}}}/7$) and 12 ns, respectively, giving a Courant–Friedrichs–Lewy number of 0.05 in soft tissue. Laterally, the spatial domain spanned 243 × 176 mm (810 × 588 grid points) to fit the transverse extent of the histotripsy transducer. The depth of the domain was set to exceed the focusing distance for the target (range: 162–230 mm, or 540–768 grid points). The outermost 8 grid points (2.4 mm) in all dimensions acted as an artificial absorbing layer to attenuate sound waves exiting the domain and prevent re-entry due to periodic Fourier boundary conditions. The simulated time duration was set depending on focusing distance (range: 120–156 *µ*s, or 10 000–13 000 time steps). Simulations were accelerated by a distributed computing cluster. Each simulation was parallelized over 30 computing cores (Xeon Gold 6154 3.00 GHz, Intel, Santa Clara, California, USA) with 60–140 GB of collective memory. The simulation execution time varied from 5–9 h based on spatial domain dimensions.

### 2.5. Acoustic property maps of the abdomen

For each target in each subject, the acoustic properties of the abdomen were modeled by rotating and interpolating the segmented anatomical images from section 2.2 to place the target in the domain at the prescribed focusing distance from the transducer and at the optimal orientation determined from section 2.3. Segmented images were interpolated from their original resolution to match the resolution of the simulation grid with the *interpn* function from MATLAB using a nearest neighbor re-sampling method. Voxels of the domain outside the body were assigned the acoustic properties of water. Voxels representing the abdomen were assigned acoustic properties given in table 2. In k-Wave simulations, large impedance differences can cause numerical errors and instability (Dourado *et al* 2022). The acoustic impedance of air (with a speed of sound of 343 m s^−1^, density of 1 kg m^−3^, and absorption coefficient 0.003 dB MHz^−1^ cm^−1^) is orders of magnitude lower than tissue and especially cortical bone (with speed of sound 3515 m s^−1^, density 1908 m s^−1^, and absorption coefficient 4.75 dB MHz^−1^ cm^−1^). Due to its very low impedance, air effectively blocks all transmission for histotripsy. Transmission through the rib bone is nonzero but does not contribute substantially to the focal pressure. Also, transmission through bone should ideally be avoided (i.e. by selectively turning off blocked portions of the aperture) to prevent heating and should not be included when calculating the treatment envelope. Therefore, in this study air and bone were both modeled as strong attenuators (i.e. non-transmitting obstructions) and were assigned the same nonphysical acoustic properties of sound speed $c$ = 3000 m s^−1^, density $\rho $ = 10 000 kg m^−3^, and ${\alpha _0}$ = 25 dB MHz^−1^ cm^−1^, which were selected to simulate strong reflection and attenuation while limiting numerical errors. As a result, the envelope calculations in this study effectively exclude any contributions to the focal pressure propagated through air or bone, which are expected to be minimal. Following assignment of acoustic properties, the 3D abdominal maps were smoothed to remove high spatial frequencies with a Blackman filter using the *smooth* function from the k-Wave Toolbox.

**Table 2.** Acoustic properties of sound path components.

Component	Sound speed (m s^−1^)	Mass density (kg m^−3^)	Absorption coefficient (dB MHz^−1^ cm^−1^)
Water^a^ (20 °C)	1483	998	0.0012
Skin	1620	1100	1.6
Fat	1430	916	0.4
Non-fat Soft tissue	1580	1050	0.5
Cartilage^b^	1650	1100	5
Acoustic obstruction (Bone/bowel/lung)^c^	3000	10 000	25

All properties were from (Duck 1990) unless otherwise noted.

^a^Sound speed (Marczak 1997), density (Jones and Harris 1992), and absorption (Pinkerton 1949).

^b^Sound speed (Goss *et al* 1978).

^c^Density and absorption were set to nonphysical values to maintain simulation stability while minimizing effective transmission.

### 2.6. Numerical acoustic source

All simulations modeled the transducer geometry described in section 2. However, representing a curved aperture in k-Wave can cause two problems. One problem is that finite-sized apertures have infinite bandwidth in the spatial frequency domain, while k-Wave represents k-space with a finite set of spatial frequencies (Firouzi *et al* 2012). Representation of an infinite bandwidth surface in band-limited k-space can distort the simulated acoustic field (Wise *et al* 2019). The other problem is that a curved surface intersects few points in discrete Cartesian space. Interpolation of curved surfaces to the discrete grid can cause staircasing errors and uneven spatial sampling (Martin *et al* 2016, Drainville *et al* 2019). Therefore, the transducer was modeled using the kWaveArray object class from the k-Wave Toolbox, which returns samples of a spatially band-limited representation of each element with appropriate scaling to compensate for variations in the sampling density (Wise *et al* 2019). Specifically, the signals transmitted or received at the grid points representing each element are weighted and averaged such that each element transmits and receives as a single, band-limited entity. In transmission, the pressure excitation signals defined by the user are re-scaled to units of rate of mass injection per unit volume and included as the source term $S$ in equation (8). For all simulations, the transducer axes were aligned with the orthogonal axes of the domain.

**Figure 4.**
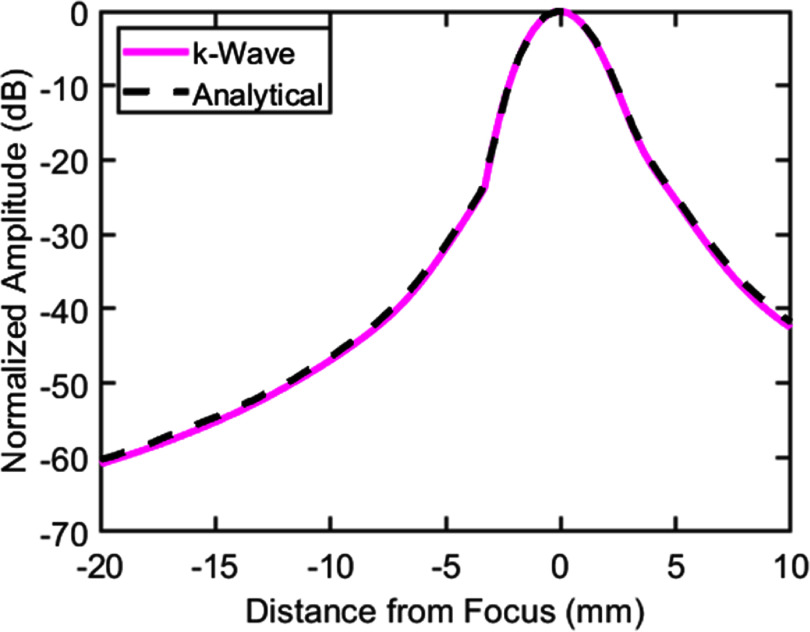
Axial profiles of the normalized rarefactional pressure amplitude in the free field for the transducer geometry in section 2.1 calculated analytically using FOCUS (blacked dashed line) and simulated in k-Wave (pink solid line).

The output of the transducer simulated in k-Wave was compared to analytical calculations for the same geometry obtained using FOCUS (McGough 2004, Kelly and McGough 2006, Chen and McGough 2008) in the free field (water). In both FOCUS and k-Wave, the transducer was simulated under transient excitation by a Hanning-windowed pulse of 750 kHz center frequency and 3 *µ*s duration. As shown in figure 4, the simulated output of the k-Wave transducer closely matched the analytically calculated focal (rarefactional) pressure amplitude (error within <.01%).

### 2.7. Simulations of acoustic propagation with and without aberration correction

**Figure 5.**
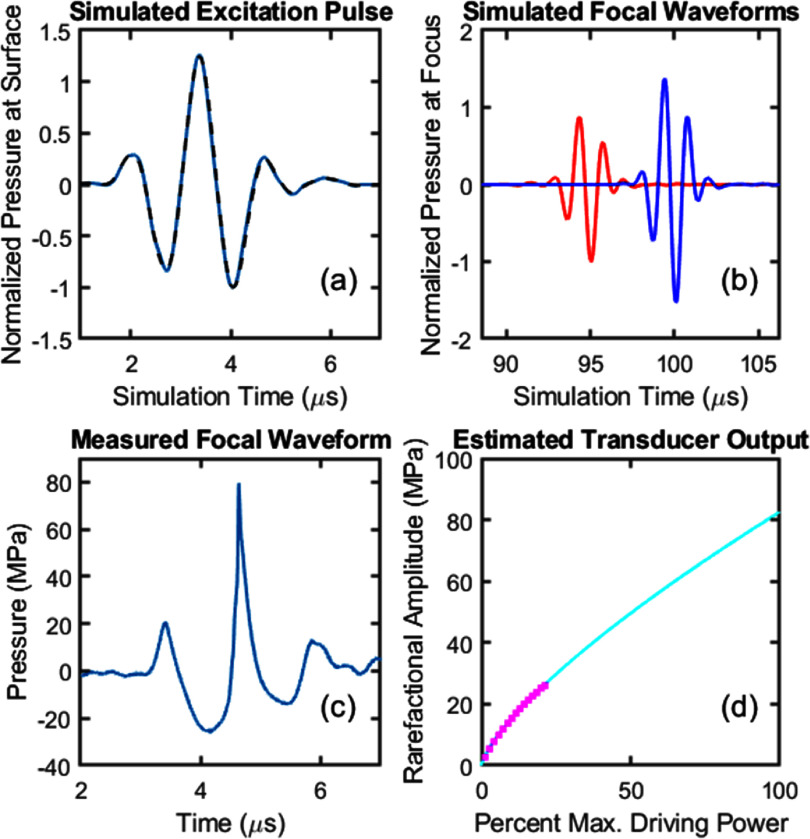
(a) Simulated excitation pulse before (blue line) and after (black dashed line) filtering. (b) Simulated focal waveforms with (blue) and without (red) aberration correction for one target in one subject. (c) Waveform measured at the focus of the transducer in the free field near the cavitation threshold. (d) Measured rarefactional amplitudes (pink dots) at low (subthreshold) transducer driving powers and fitted function (cyan line) used to estimate the rarefactional amplitude at maximum driving power.

At each target in each subject, 3 simulations were performed. One simulation placed a point source emitting an impulse at the target and received the resulting time-varying pressure with the transducer array elements (following the ‘time reversal’ approach for aberration correction (Fink 1992)). Relative travel times to each of the elements were determined by detecting the peak amplitude of the arriving impulse. The other two simulations set all elements to transmit a 2-cycle pulse set from a waveform measured at the focus of the physical transducer described in section 2 and analytically backpropagated to the transducer surface. High frequencies (>2 MHz) in the backpropagated waveform were attenuated prior to simulation using the k-Wave *filterTimeSeries* function to apply a low-pass finite impulse response filter with 60 dB attenuation in the stopband, a transition width of 120 ns, and a cutoff frequency of 1.58 MHz, ensuring that the grid spacing was <1/3 of the smallest wavelength. The backpropagated waveform is shown before and after filtering in figure 5(a).

The first of the two ‘transmit’ simulations had no aberration correction. For targets at the geometric focus of the transducer, all elements transmitted simultaneously. For targets requiring an extended focusing distance (i.e. electronic focal steering + no aberration correction), pulse transmission was differentially delayed across elements to focus at the target assuming a homogeneous sound speed of 1540 m s^−1^. For all targets (i.e. with or without steering), a second ‘transmit’ simulation modeled time reversal aberration correction by delaying the transmission of each element to compensate for travel time differences detected from the ‘receive’ simulation. For both transmit simulations, the maximum rarefactional amplitude was recorded at each grid point to obtain the focal pressure (maximum rarefactional pressure in the field).

To estimate the treatment envelope, the simulated focal pressures were used to estimate a maximum obtainable focal pressure for each target. First, the focal pressures simulated both with and without aberration correction were divided by the focal pressure simulated for the same transducer and excitation in the free field (water). These ratios of focal pressure in the body to focal pressure in the free field were then scaled by multiplying by the estimated maximum free field rarefactional amplitude of the physical transducer from our lab of 80 MPa. The maximum rarefactional pressure was estimated by measuring output from the physical transducer at subthreshold (<26 MPa) rarefactional amplitudes with a fiber optic hydrophone (HFO-690, Onda, Sunnyvale, CA) and then extrapolating via a polynomial fit (Vlaisavljevich *et al* 2017). A waveform measured at the focus at a driving power level near the cavitation threshold is shown in figure 5(c). The rarefactional amplitudes measured at all driving power levels are shown with the polynomial fit in figure 5(d). The fitted polynomial function matched well with subthreshold measurements and predicted array output more conservatively than a linear relationship with driving power. However, it should be noted that the true maximum rarefactional pressure cannot be directly measured due to cavitation, nor predicted numerically for nonlinear conditions, and is thus unknown. The uncertainty in the estimate of maximum transducer output, along with the use of the linear acoustic propagation model, is a limitation of this study which is discussed further in section 4.

## 3. Results

**Figure 6.**
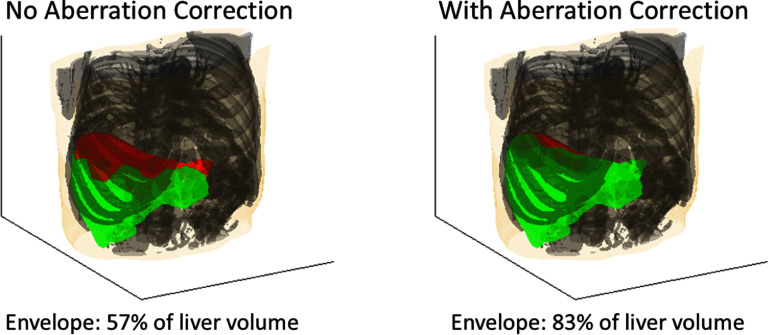
Map of the treatment envelope in the liver of one subject, both without and with aberration correction. Red denotes regions where focal pressure is insufficient to perform histotripsy. Green denotes regions where histotripsy can be performed.

Aberration correction increased the focal pressure throughout the liver to expand the treatment envelope. Figure 6 maps the treatment envelope for the liver of one subject both with and without aberration correction. In green regions of the map, the linearly predicted focal pressure exceeded the cavitation threshold. Red regions indicate where the focal pressure was below the threshold. Without aberration correction, the treatment envelope was confined to more shallow, subcostal regions of the liver and comprised 57% of the total liver volume. Couinaud segments II and VII were entirely excluded from the treatable region. With aberration correction, the treatment envelope expanded into deeper, more cranial regions, increasing to 83% of the liver volume. Aberration correction increased the treatable portions of Couinaud segments II and VII to 100% and 25% of the segment volumes, respectively.

**Figure 7.**
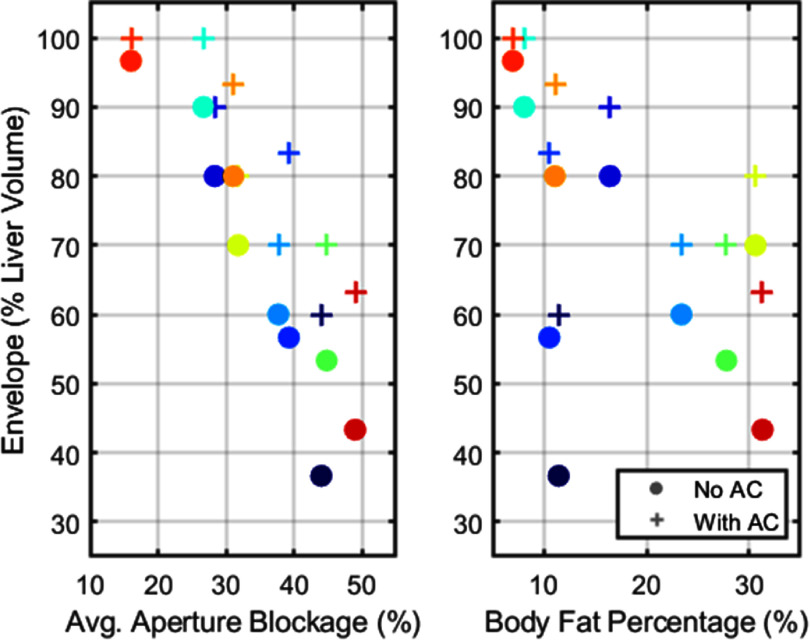
Scatterplots of the treatment envelope (% volume of liver with maximum attainable focal pressure ⩾26 MPa) for each anatomical subject versus average aperture blockage (left) and estimated body fat percentage (right). Different colors represent different anatomical subjects.

By increasing the focal pressure, aberration correction expanded the histotripsy treatment envelope for all subjects. Figure 7 shows the size of the treatment envelope (percent volume of the liver with amplitude ⩾26 MPa) for each subject without (circle markers) and with (cross markers) correction as a function of aperture blockage (left) and body fat percentage (right). On average, aberration correction increased the volume of the liver with focal pressure ⩾26 MPa from 67 ± 20% to 81 ± 15%. Across subjects, the treatment envelope ranged from 37% to 97% of the liver without aberration correction and from 60% to 100% of the liver with aberration correction. Subjects with larger average blockage of the aperture tended to have more limited treatment envelopes (*R*^2^ = 0.90 without aberration correction, *R*^2^ = 0.80 with aberration correction, using standard linear regression), whereas the relationship between the treatment envelope and the body fat percentage was less clear (*R*^2^ = 0.24 without aberration correction, *R*^2^ = 0.39 with aberration correction). The two largest expansions of the treatment envelope (from 37% and 57% without aberration correction to 60% and 83% with aberration correction, respectively) were for subjects with highly obstructed livers (44% and 40% average aperture blockage, respectively). The smallest three expansions (70%, 90%, and 97% without aberration correction to 80%, 100%, and 100% with aberration correction) were for subjects with relatively low average aperture blockage (32%, 27%, and 16%, respectively).

**Figure 8.**
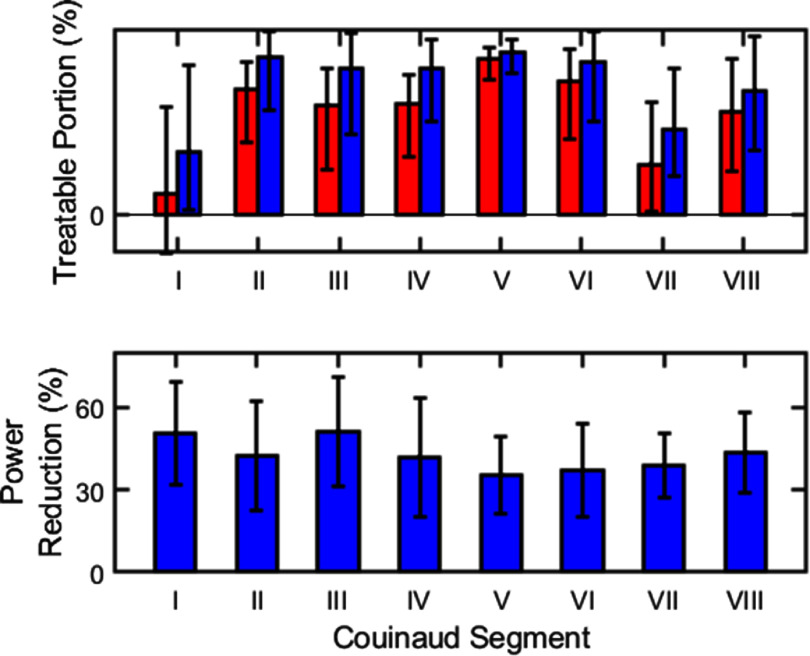
(Top): average treatable fraction of each liver Couinaud segment with (blue) and without (red) aberration correction. (Bottom): percentage reduction in input acoustic power that would be required to perform histotripsy with aberration correction. Bars denote average values over subjects and targets. Error bars show the standard deviation.

The effect of aberration correction varied across liver segments. As shown in figure 8 (top panel), aberration correction substantially expanded the treatment envelope for Couinaud segments I and VII. Without aberration correction, only 13% and 30% of segments I and VII were treatable on average, respectively. Aberration correction increased these portions to 38 and 50%, respectively. For Couinaud segments II and V, large portions (75% and 93%, respectively) were treatable on average without aberration correction. With correction, the average treatable portions increased to 94% and 97%, respectively. For the remaining segments (III, IV, VI, and VIII), the average treatable portion ranged from 61%–80% without correction and from 74%–91% with correction. Sample standard deviations were large due to variation across subjects and lower sampling for smaller segments.

In addition to expanding the treatment envelope, aberration correction reduced the input acoustic power that would be required to perform histotripsy throughout the liver. Specifically, by increasing the focal pressure achieved for a given acoustic input, aberration correction reduced the input power that would be required to generate cavitation at a given target. This power reduction was estimated as $100\% \times \left( {1 - { }\frac{{P_{{\text{NAC}}}^2}}{{P_{{\text{AC}}}^2}}} \right)$, where ${P_{{\text{NAC}}}}$ and ${P_{{\text{AC}}}}$ are the maximum achievable focal pressure with and without aberration correction, respectively. The average power reduction for each Couinaud segment is given in figure 8 (bottom panel). Across all segments, aberration correction reduced the required input power by 43% on average (min: 35%, max: 51%). Within segments, the power reduction varied substantially. The per-segment sample standard deviation ranged from ±12% (segment VII) to ±22% (segment IV).

**Figure 9.**
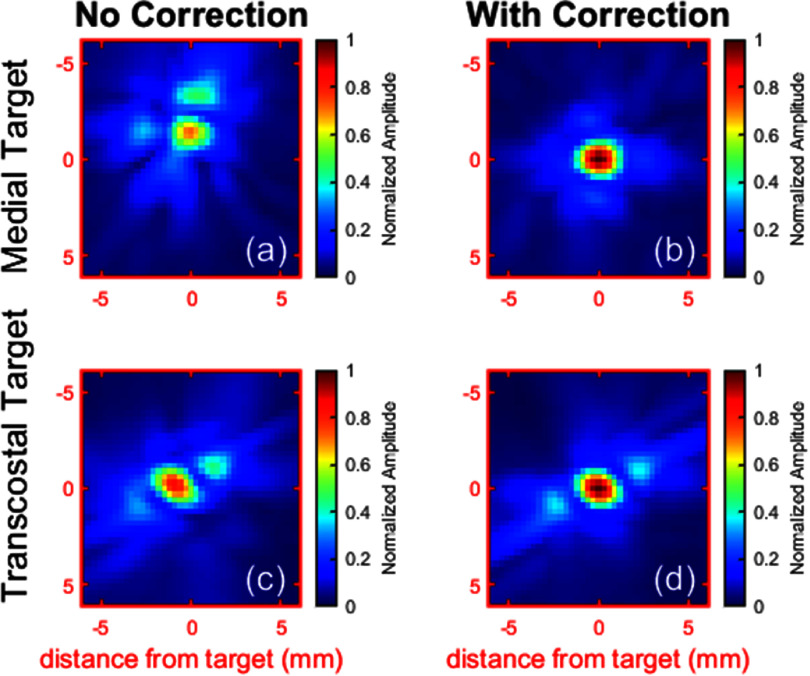
Cross sections of the linearly predicted rarefactional pressure distribution at the focus in the transverse plane of the transducer with and without aberration correction for a medial target (a), (b) in the liver and a transcostal target (c), (d). Each pair of pressure maps is normalized to the corrected maximum.

Aberration correction also improved focus quality and targeting accuracy. As illustrated in figures 9 (a) and (b), aberration correction transformed the linearly predicted focus for most targets from multiple lobes of irregular shape to a single, regular, ellipsoidal lobe. For transcostal targets where the ribs formed a grating pattern across the aperture, aberration correction did not reduce the number of focal lobes (from 3) but did increase the amplitude of the central lobe relative to the 2 side lobes (see figures 9(c) and (d)). Across all subjects, aberration correction reduced the focal volume (3D region where the rarefactional amplitude exceeded 50% of the spatial maximum) by 58% (from 48 ± 40 mm^3^ to 20 ± 10 mm^3^) for targets within the transducer focal length and by 33% (from 192 ± 70 mm^3^ to 128 ± 63 mm^3^) for targets beyond the focal length. Aberration correction also reduced targeting error, decreasing the average focal shift (Euclidian distance between the maximum pressure location and the target) across all subjects and targets from 3.8 ± 2.2 mm to 0.2 ± 0.3 mm (within the spatial resolution of the simulation grid).

**Figure 10.**
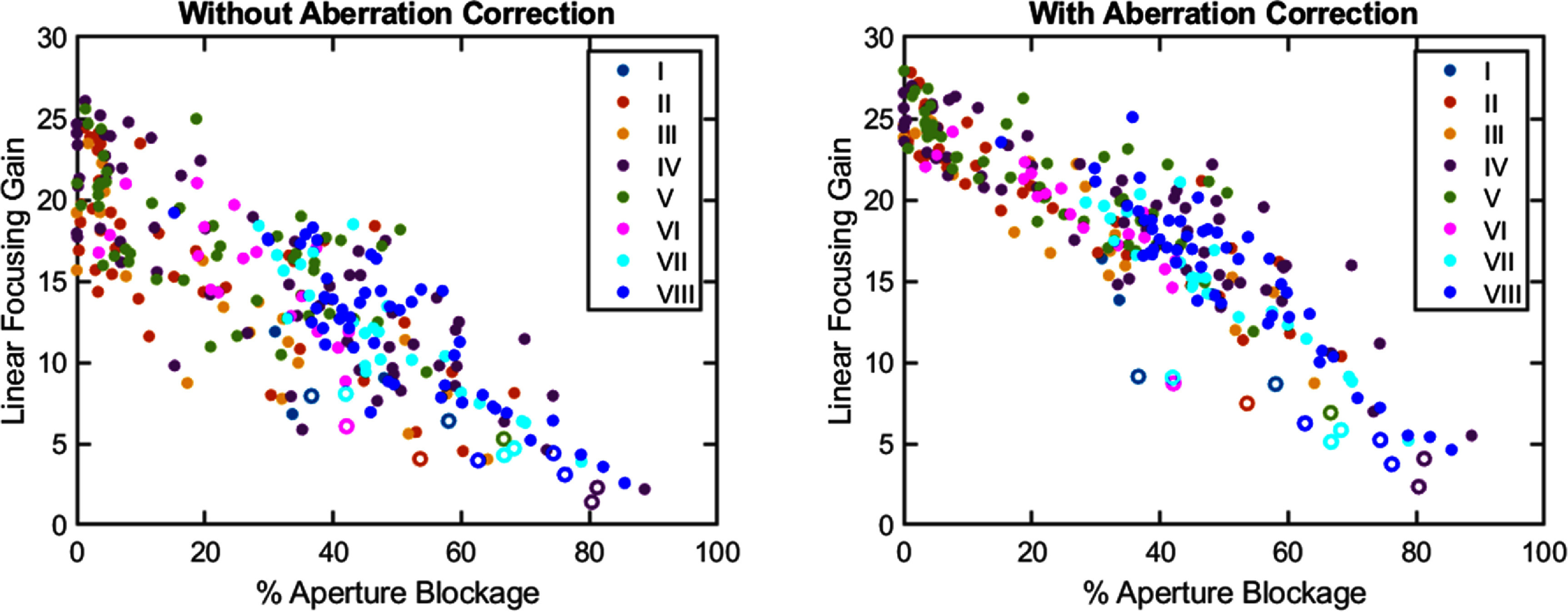
Scatterplots of the linear focusing gain (ratio of the maximum rarefactional amplitude at the focus to the maximum rarefactional amplitude at the transducer surface) versus percent aperture blockage per target, both without (left) and with (right) aberration correction. In both plots, filled markers denote targets at the focal length of the histotripsy array. Ring (non-filled) markers denote targets requiring electronic focal steering to focus beyond the focal length. Marker color denotes the liver Couinaud segment (I–VIII).

Aberration correction increased the linear focusing gain for all targets and subjects. Figure 10 shows the linear focusing gain (ratio of the focal pressure to the rarefactional amplitude at the transducer surface) against aperture blockage for all targets and subjects both with and without aberration correction. As expected, the linear focusing gain decreased with increasing aperture blockage. Thus, several targets in cranial regions (Couinaud segments IV, VII and VIII) had low focusing gain even after aberration correction. The focusing gain decreased further when electronically steering to focus beyond the focal length of the transducer. All targets deeper than the focal length (denoted by open ring markers in figure 10) had very low focusing gain (<10) even after aberration correction. For equivalent aperture blockage, targets requiring electronic focal steering always had lower focusing gain after aberration correction than targets at the focal length.

## 4. Discussion

Aberration correction could substantially expand the treatment envelope for histotripsy in the liver. For a phased array with similar dimensions and acoustic output to the current clinical device, aberration correction expanded the linearly predicted treatment envelope from 67% to 81% of the liver volume on average, extending the treatable region from shallow, caudal segments of the liver to deeper, more cranial segments (see figures 6 and 8). Clinical trials have thus far restricted histotripsy treatment to tumors in accessible, less obstructed segments of the liver (II–VI), excluding segments I, VII, and VIII (Ziemlewicz *et al* 2025). Here, aberration correction increased the average treatable portion of segments I and VII by 200% and 75% (relative to the treatable volume without correction). The results of this study indicate that, as clinical use of histotripsy advances, aberration correction will be essential to treat more challenging locations in the liver and thereby help more patients access histotripsy therapy.

Aberration correction will be more critical to enable histotripsy treatment for some patients than others. For one anatomical subject in this study, aberration correction expanded the treatment envelope by 63%, whereas for another subject, aberration correction only expanded the treatment envelope by 11%. Such differences across subjects likely result from anatomical variations. The results in figure 7 indicate that patients with livers highly obstructed by ribs tend to have more restricted treatment envelopes than patients with less blockage of the liver and thus may be more likely to require aberration correction for histotripsy treatment. Notably, the size of the treatment envelope did not correlate strongly with body fat percentage, suggesting that the treatment envelope depends more on acoustic access to the liver than body composition.

In addition to expanding the treatment envelope, aberration correction improves the safety and efficiency of histotripsy treatment at all locations in the liver. As shown in figure 8 (bottom panel), aberration correction reduced the input acoustic power that would be required to generate cavitation at all locations by 43% on average. By decreasing the acoustic power delivered to intervening tissues, aberration correction could help reduce tissue heating, alleviating the need for cooling time pauses and enabling safer, faster treatments. Aberration correction also reduced the size of the focal volume, improved focal shape, and eliminated targeting error from focal shift. Without correction, the focus is often diffuse, irregularly shaped, and off-target, limiting the precision of histotripsy ablations (see figure 9(a)). By creating a sharp, regularly shaped focus at the target (see figure 9(b)), aberration correction could enhance the ability to plan and generate precise lesions in the liver. Thus far, clinical trials have primarily treated large tumors (median diameter: 1.3 cm (Ziemlewicz *et al* 2025)) with dimensions much greater than the focal volume and the focal shift, alleviating the requirements on focal shape and targeting accuracy for precise ablation. As clinicians begin to treat smaller tumors with finer ablation margins, aberration correction may be necessary to precisely control the focus and the lesion volume.

Aberration correction will not be the only step necessary to expand the treatment envelope for transabdominal histotripsy. For deep, cranial regions of the liver, additional challenges limit the safety and feasibility of histotripsy treatment. Couinaud segment I surrounds and abuts critical anatomical structures of the liver, including major blood and biliary vessels like the inferior vena cava and those of the liver hilum (portal vein, hepatic artery, and hepatic duct). Concerns about damaging these structures have helped motivate the exclusion of segment I from clinical trials. However, studies have shown that blood vessels and bile ducts are more resistant to histotripsy damage than liver parenchyma, suggesting that histotripsy may be able to destroy tumors in this segment while sparing these critical structures (Lake *et al* 2008, Vlaisavljevich *et al* 2014, 2015, Elliott and Simon 2023). Further pre-clinical research on the capability for selective damage will be required to validate the safety of histotripsy ablations in segment I. For transcostal segments VII and VIII, the safety of histotripsy treatments is limited by intervening ribs, which strongly absorb ultrasound energy, causing unsafe heating over extended treatment times. Correction of phase errors can reduce heating by lowering the power input required to perform histotripsy but does not altogether prevent the delivery of acoustic energy through the ribs. Therefore, to ensure safety, transcostal histotripsy treatments will likely require not only phase correction but also an amplitude apodization, where transducer elements whose beams intersect ribs are detected and selectively de-activated to prevent transmission through ribs. Previous work has shown that elements blocked by ribs can be detected either numerically (using a pre-treatment image spatially registered to the patient body and the transducer (Liu *et al* 2007, 2010, Quesson *et al* 2010, Gélat *et al* 2012, Qiao *et al* 2013, de Senneville *et al* 2016)) or acoustically (by receiving acoustic signals with the therapy array and analyzing their amplitude (Aubry *et al* 2008, Cochard *et al* 2009, 2011, Ballard *et al* 2010, Bobkova *et al* 2010, Marquet *et al* 2011, Jakovljevic *et al* 2017, Zubair and Dickinson 2021)). Elements detected as blocked can then be de-activated to reduce heating at the ribs. De-activation of blocked elements (specifically those which are only partially blocked by ribs) may decrease the maximum obtainable focal pressure for certain treatment locations. However, for many transcostal locations, rib heating is a greater concern than pressure amplitude, effectively restricting the treatment envelope further than the volume determined in this study. Therefore, strategies for amplitude apodization will likely be a critical complement to phase aberration correction in enabling histotripsy treatment in transcostal regions of the liver.

Like the ribs, the lung and bowels limit the treatment envelope by blocking ultrasound and introducing safety concerns (i.e. by increasing the risk of cavitation at an air-tissue interface). These challenges can be partially alleviated by clinical strategies. To maximize acoustic access and prevent air pockets from intersecting the ultrasound beam, it is critical that bowel gas is reduced prior to treatment (e.g. through fasting and medication). Blockage by the lung is more challenging to address but could be avoided in some cases by strategically positioning the patient, as abdominal organs can shift with posture.

The results of this study indicate that the treatment envelope is also limited by the focal length of the transducer. Here, for 14% of the liver on average (min: 0%, max: 27%) the center of curvature of the transducer could not be placed at the target without intersecting the skin. For most of these targets, (9% of the liver on average), the required focusing depth exceeded the electronic steering range of the array (3 cm). For the remaining targets, the focus was electronically steered between 2 and 3 cm beyond the focal length. Due to combined loss from electronic steering, attenuation, and aberration, these targets had very low linear focusing gains and were all determined to be untreatable with histotripsy. It should be noted that this study may overestimate the number of targets requiring electronic focal steering by using anatomical images obtained without compression of the abdomen. In real histotripsy treatment scenarios, the abdomen is compressed by the water bolus used to facilitate acoustic coupling between the transducer and the body. This compression partially flattens the abdomen and may bring more of the liver within the focal length of the transducer. However, the water bolus also pushes the liver up towards the head and under the ribs, increasing blockage and potentially decreasing the treatment envelope. In future studies, we will assess how compression of the abdomen by the water bolus affects liver blockage, targeting depth, and the extent of the treatment envelope.

Also, in this study phase corrections were derived by receiving signals from a point source placed at the target, permitting simultaneous elimination of the focal shift and increase of focal pressure. In practice, point-like sources are not available at the target. Instead, correction of both focal shift and focal pressure requires a 2-step approach, where first *a priori* knowledge of the ultrasound path (e.g. from medical images) is used to calculate a preliminary phase correction that reduces focal shift, and second, acoustic signals are received from the corrected focal location to refine the phase correction and restore substantial focal pressure (Gateau *et al* 2010). Lu *et al* demonstrated the 2-step aberration correction approach for transcranial histotripsy, reducing the average targeting error from 0.8 mm to 0.3 mm and restoring 87% of the focal pressure lost to aberration (2022). For transabdominal histotripsy, the 2 steps of the 2-step aberration correction method have not yet been demonstrated together but have been demonstrated separately. Wagner *et al* used images from a cone beam computed tomography system spatially registered to the therapy transducer to correct focal shift for transabdominal histotripsy in porcine subjects, achieving an average targeting accuracy of <1.5 mm (Wagner *et al* 2023). Methods to restore focal pressure using received acoustic signals (either backscattered signals from ultrasound pulses (Thomas *et al* 2021, 2022) or acoustic emissions from cavitation bubbles (Yeats *et al* 2023, 2024)) have been shown *in vitro* with excised porcine abdominal wall to recover >95% of focal pressure lost to aberration and have been validated in porcine subjects *in vivo*. If combined into a 2-step correction approach, these strategies could foreseeably achieve similar efficacy to the phase corrections simulated here, suggesting that aberration correction is feasible and could substantially improve transabdominal histotripsy therapy.

It is important to note that the predictions of the treatment envelope reported here rely on linear simulations of histotripsy focusing. In intrinsic threshold histotripsy, generation of cavitation depends on the amplitude of the rarefactional pressure phase. The rarefactional phase does not narrow sharply like the compressional phase under nonlinear distortion (Yuldashev *et al* 2013). Also, intrinsic threshold histotripsy typically requires very low f-number (⩽1) transducers, which restrict nonlinear propagation effects to a small region around the focus (<5 mm for the transducer simulated here) (Yeats *et al* 2022). Attenuation by tissue further reduces the amplitude of the pre-focal converging wavefront. These observations suggest that linear simulations can suitably model the wavefront degradation resulting from aberration over most of the ultrasound path. At the focus, the rarefactional phase distorts (broadens, weakens, and shifts pre-focally) relative to linear conditions. For rarefactional amplitudes near the cavitation threshold, these differences are unlikely to substantially alter the predicted field. For amplitudes beyond the cavitation threshold, the linearly predicted rarefactional pressure can greatly exceed nonlinear predictions, strongly limiting the utility of the linear model (Bessonova *et al* 2009).

Another limitation of the study is that the maximum attainable rarefactional pressure of the physical transducer was estimated by measuring output at low (sub-cavitation) driving power with a hydrophone and then extrapolating to the highest power setting via a polynomial fit (Vlaisavljevich *et al* 2017). This approach was necessary due to cavitation at the focus above the threshold but likely overestimates the maximum attainable rarefactional pressure amplitude, which saturates (rapidly reaches a plateau) with increasing pressure for highly focused transducers (Bessonova *et al* 2009). Consequently, this study likely overestimates the focal pressure in the liver and the treatment envelope. Despite these limitations, the relative values of corrected and uncorrected focal pressure in this study indicate that aberration correction can substantially improve transabdominal histotripsy therapy and expand the treatment envelope in the liver. Similar studies could help to assess the effect of aberration correction for other abdominal organs like kidney and pancreas that are now undergoing phase I clinical trials for histotripsy treatment. For the retroperitoneal kidney and the deep-seated pancreas, it will be important to additionally consider the effects of patient positioning, which could be strategically modified to enhance the acoustic window (i.e. by shifting the organ relative to surrounding blockages).

## 5. Conclusion

The treatment envelope for histotripsy in the human liver was assessed both with and without aberration correction using a linear numerical model for a phased array with similar dimensions to the current (Histosonics Edison®) clinical device. Simulations indicate that aberration correction can substantially expand the treatment envelope (from 67% to 81% of the liver for intrinsic threshold histotripsy), increasing focal pressure throughout the liver to enable histotripsy in deeper, more cranial liver regions and in a broader population of patients. Aberration correction could also increase the safety and precision of transabdominal histotripsy treatments by reducing the input power required to generate cavitation (by 43%, on average) and by improving focusing quality. Together, these results suggest that aberration correction will be critical to improve treatment efficacy and safety and to allow more patients to access non-invasive, non-thermal, and non-ionizing histotripsy therapy.

## Data Availability

The data that support the findings of this study are openly available at the following URL/DOI: https://doi.org/10.7302/cbbn-zh54 (Yeats n.d.).

## References

[pmbae222cbib1] Aubry J-F, Pernot M, Marquet F, Tanter M, Fink M (2008). Transcostal high-intensity-focused ultrasound: ex vivo adaptive focusing feasibility study. Phys. Med. Biol..

[pmbae222cbib2] Ballard J R, Casper A J, Wan Y, Ebbini E S (2010). Adaptive transthoracic refocusing of dual-mode ultrasound arrays. IEEE Trans. Biomed. Eng..

[pmbae222cbib3] Bancel T, Houdouin A, Annic P, Rachmilevitch I, Shapira Y, Tanter M, Aubry J-F (2021). Comparison between ray-tracing and full-wave simulation for transcranial ultrasound focusing on a clinical system using the transfer matrix formalism. IEEE Trans. Ultrason. Ferroelectr. Freq. Control.

[pmbae222cbib4] Bawiec C R (2021). A prototype therapy system for boiling histotripsy in abdominal targets based on a 256-element spiral array. IEEE Trans. Ultrason. Ferroelectr. Freq. Control.

[pmbae222cbib5] Bessonova O V, Khokhlova V A, Bailey M R, Canney M S, Crum L A (2009). Focusing of high power ultrasound beams and limiting values of shock wave parameters. Acoust. Phys..

[pmbae222cbib6] Bobina A S, Rosnitskiy P B, Khokhlova T D, Yuldashev P V, Khokhlova V A (2021). Effect of abdominal wall inhomogeneities on the focusing of an ultrasonic beam at different positions of the transducer. Bull. Russ. Acad. Sci. Phys..

[pmbae222cbib7] Bobkova S, Gavrilov L, Khokhlova V, Shaw A, Hand J (2010). Focusing of high-intensity ultrasound through the rib cage using a therapeutic random phased array. Ultrasound Med. Biol..

[pmbae222cbib8] Chen D, McGough R J (2008). A 2D fast near-field method for calculating near-field pressures generated by apodized rectangular pistons. J. Acoust. Soc. Am..

[pmbae222cbib9] Clark K (2013). The cancer imaging archive (TCIA): maintaining and operating a public information repository. J. Digit. Imaging.

[pmbae222cbib10] Cochard E, Aubry J F, Tanter M, Prada C (2011). Adaptive projection method applied to three-dimensional ultrasonic focusing and steering through the ribs. J. Acoust. Soc. Am..

[pmbae222cbib11] Cochard E, Prada C, Aubry J F, Fink M (2009). Ultrasonic focusing through the ribs using the DORT method. Med. Phys..

[pmbae222cbib12] Couinaud C (2000). Liver anatomy: portal (and suprahepatic) or biliary segmentation. Digestive Surg..

[pmbae222cbib13] de Greef M, Schubert G, Wijlemans J W, Koskela J, Bartels L W, Moonen C T W, Ries M (2015). Intercostal high intensity focused ultrasound for liver ablation: the influence of beam shaping on sonication efficacy and near-field risks. Med. Phys..

[pmbae222cbib14] de Senneville B D, Moonen C, Ries M, Escoffre J-M, Bouakaz A (2016). MRI-guided HIFU methods for the ablation of liver and renal cancers. Therapeutic Ultrasound, Advances in Experimental Medicine and Biology.

[pmbae222cbib15] Dillon C R, Farrer A, McLean H, Almquist S, Christensen D, Payne A (2018). Experimental assessment of phase aberration correction for breast MRgFUS therapy. Int. J. Hyperth..

[pmbae222cbib16] Dourado T C, Alvarenga A V, Peters F C, Mansur W J, Costa-Félix R P B (2022). Simultaneous use of pulse-echo and through-transmission methods in determining a combined reflection coefficient. Appl. Acoust..

[pmbae222cbib17] Drainville R A, Curiel L, Pichardo S (2019). Superposition method for modelling boundaries between media in viscoelastic finite difference time domain simulations. J. Acoust. Soc. Am..

[pmbae222cbib18] Duck F A, Duck F A (1990). Acoustic properties of tissue at ultrasonic frequencies. Physical Properties of Tissues.

[pmbae222cbib19] Elliott J, Simon J C (2023). Histotripsy bubble dynamics in elastic, anisotropic tissue-mimicking phantoms. Ultrasound Med. Biol..

[pmbae222cbib20] Fan X, Hynynen K (1992). The effect of wave reflection and refraction at soft tissue interfaces during ultrasound hyperthermia treatments. J. Acoust. Soc. Am..

[pmbae222cbib21] Fink M (1992). Time reversal of ultrasonic fields. I. Basic principles. IEEE Trans. Ultrason. Ferroelectr. Freq. Control.

[pmbae222cbib22] Firouzi K, Cox B T, Treeby B E, Saffari N (2012). A first-order k-space model for elastic wave propagation in heterogeneous media. J. Acoust. Soc. Am..

[pmbae222cbib23] Flax S W, O’Donnell M (1988). Phase-aberration correction using signals from point reflectors and diffuse scatterers: basic principles. IEEE Trans. Ultrason. Ferroelectr. Freq. Control.

[pmbae222cbib24] Gao J, Cochran S, Huang Z (2014). Ultrasound beam distortion and pressure reduction in transcostal focused ultrasound surgery. Appl. Acoust..

[pmbae222cbib25] Gateau J, Marsac L, Pernot M, Aubry J-F, Tanter M, Fink M (2010). Transcranial ultrasonic therapy based on time reversal of acoustically induced cavitation bubble signature. IEEE Trans. Biomed. Eng..

[pmbae222cbib26] Gélat P, Ter Haar G, Saffari N (2012). The optimization of acoustic fields for ablative therapies of tumours in the upper abdomen. Phys. Med. Biol..

[pmbae222cbib27] Goss S A, Johnston R L, Dunn F (1978). Comprehensive compilation of empirical ultrasonic properties of mammalian tissues. J. Acoust. Soc. Am..

[pmbae222cbib28] Hall T L, Hempel C R, Sabb B J, Roberts W W (2010). Acoustic access to the prostate for extracorporeal ultrasound ablation. J. Endourol..

[pmbae222cbib29] Hynynen K, Jones R M (2016). Image-guided ultrasound phased arrays are a disruptive technology for non-invasive therapy. Phys. Med. Biol..

[pmbae222cbib30] Jakovljevic M, Pinton G F, Dahl J J, Trahey G E (2017). Blocked elements in 1-D and 2-D arrays—part I: detection and basic compensation on simulated and *in vivo* targets. IEEE Trans. Ultrason. Ferroelectr. Freq. Control.

[pmbae222cbib31] Jing Y, Meral F C, Clement G T (2012). Time-reversal transcranial ultrasound beam focusing using a k-space method. Phys. Med. Biol..

[pmbae222cbib32] Jones F E, Harris G L (1992). ITS-90 density of water formulation for volumetric standards calibration. J. Res. Natl Inst. Stand. Technol..

[pmbae222cbib33] Jones R M, Hynynen K (2015). Comparison of analytical and numerical approaches for CT-based aberration correction in transcranial passive acoustic imaging. Phys. Med. Biol..

[pmbae222cbib34] Kelly J F, McGough R J (2006). A time-space decomposition method for calculating the nearfield pressure generated by a pulsed circular piston. IEEE Trans. Ultrason. Ferroelectr. Freq. Control.

[pmbae222cbib35] Khokhlova T D (2019). Pilot *in vivo* studies on transcutaneous boiling histotripsy in porcine liver and kidney. Sci. Rep..

[pmbae222cbib36] Khokhlova V A, Fowlkes J B, Roberts W W, Schade G R, Xu Z, Khokhlova T D, Hall T L, Maxwell A D, Wang Y-N, Cain C A (2015). Histotripsy methods in mechanical disintegration of tissue: towards clinical applications. Int. J. Hyperth..

[pmbae222cbib37] Kim Y, Vlaisavljevich E, Owens G E, Allen S P, Cain C A, Xu Z (2014). *In vivo* transcostal histotripsy therapy without aberration correction. Phys. Med. Biol..

[pmbae222cbib38] Kisting M A (2024). Safety and efficacy of histotripsy delivery through overlying gas-filled small bowel in an ex vivo swine model. Int. J. Hyperth..

[pmbae222cbib39] Knott E A (2021). Transcostal histotripsy ablation in an *in vivo* acute hepatic porcine model. CardioVascular Interventional Radiol..

[pmbae222cbib40] Knott E A (2023). A comparison study of microwave ablation vs. histotripsy for focal liver treatments in a swine model. Eur. Radiol..

[pmbae222cbib41] Lake A M, Xu Z, Wilkinson J E, Cain C A, Roberts W W (2008). Renal ablation by histotripsy—does it spare the collecting system?. J. Urol..

[pmbae222cbib42] Leung S A, Moore D, Webb T D, Snell J, Ghanouni P, Butts Pauly K (2021). Transcranial focused ultrasound phase correction using the hybrid angular spectrum method. Sci. Rep..

[pmbae222cbib43] Lin K, Kim Y, Maxwell A D, Wang T, Hall T L, Xu Z, Fowlkes J B, Cain C A (2014). Histotripsy beyond the intrinsic cavitation threshold using very short ultrasound pulses: microtripsy. IEEE Trans. Ultrason. Ferroelectr. Freq. Control.

[pmbae222cbib44] Liu H-L, Chang H, Chen W-S, Shih T-C, Hsiao J-K, Lin W-L (2007). Feasibility of transrib focused ultrasound thermal ablation for liver tumors using a spherically curved 2D array: a numerical study. Med. Phys..

[pmbae222cbib45] Liu H-L, Hsu C-L, Huang S-M, Hsi Y-W (2010). Focal beam distortion and treatment planning for transrib focused ultrasound thermal therapy: a feasibility study using a two-dimensional ultrasound phased array. Med. Phys..

[pmbae222cbib46] Lu N, Hall T L, Sukovich J R, Choi S W, Snell J, McDannold N, Xu Z (2022). Two-step aberration correction: application to transcranial histotripsy. Phys. Med. Biol..

[pmbae222cbib47] Lu N, Yeats E M, Sukovich J R, Hall T L, Pandey A S, Xu Z (2024). Treatment envelope of transcranial histotripsy: challenges and strategies to maximize the treatment location profile. Phys. Med. Biol..

[pmbae222cbib48] Macoskey J J, Hall T L, Sukovich J R, Choi S W, Ives K, Johnsen E, Cain C A, Xu Z (2018). Soft-tissue aberration correction for histotripsy. IEEE Trans. Ultrason. Ferroelectr. Freq. Control.

[pmbae222cbib49] Magnier C, Kwiecinski W, Escudero D S, Amis G, Goudot G, Mousseaux E, Messas E, Pernot M (2025). A 3D numerical model of ultrasonic transthoracic propagation for cardiac focused ultrasound therapy. IEEE Trans. Biomed. Eng..

[pmbae222cbib50] Manuel T J (2025). Ultra-short time-echo based ray tracing for transcranial focused ultrasound aberration correction in human calvaria. Phys. Med. Biol..

[pmbae222cbib51] Marczak W (1997). Water as a standard in the measurements of speed of sound in liquids. J. Acoust. Soc. Am..

[pmbae222cbib52] Marquet F, Aubry J F, Pernot M, Fink M, Tanter M (2011). Optimal transcostal high-intensity focused ultrasound with combined real-time 3D movement tracking and correction. Phys. Med. Biol..

[pmbae222cbib53] Martin E, Ling Y T, Treeby B E (2016). Simulating focused ultrasound transducers using discrete sources on regular Cartesian grids. IEEE Trans. Ultrason. Ferroelectr. Freq. Control.

[pmbae222cbib54] Maxwell A D, Cain C A, Hall T L, Fowlkes J B, Xu Z (2013). Probability of cavitation for single ultrasound pulses applied to tissues and tissue-mimicking materials. Ultrasound Med. Biol..

[pmbae222cbib55] McGough R J (2004). Rapid calculations of time-harmonic nearfield pressures produced by rectangular pistons. J. Acoust. Soc. Am..

[pmbae222cbib56] Mendiratta-Lala M (2024). The #HOPE4LIVER single-arm pivotal trial for histotripsy of primary and metastatic liver tumors. Radiology.

[pmbae222cbib57] Mougenot C, Tillander M, Koskela J, Köhler M O, Moonen C, Ries M (2012). High intensity focused ultrasound with large aperture transducers: a MRI based focal point correction for tissue heterogeneity. Med. Phys..

[pmbae222cbib58] Ng G C, Worrell S S, Freiburger P D, Trahey G E (1994). A comparative evaluation of several algorithms for phase aberration correction. IEEE Trans. Ultrason. Ferroelectr. Freq. Control.

[pmbae222cbib59] Nock L, Trahey G E, Smith S W (1989). Phase aberration correction in medical ultrasound using speckle brightness as a quality factor. J. Acoust. Soc. Am..

[pmbae222cbib60] Pinkerton J M M (1949). The absorption of ultrasonic waves in liquids and its relation to molecular constitution. Proc. Phys. Soc. B.

[pmbae222cbib61] Qiao S, Shen G, Bai J, Chen Y (2013). Transcostal high-intensity focused ultrasound treatment using phased array with geometric correction. J. Acoust. Soc. Am..

[pmbae222cbib62] Quesson B, Merle M, Köhler M O, Mougenot C, Roujol S, de Senneville B D, Moonen C T (2010). A method for MRI guidance of intercostal high intensity focused ultrasound ablation in the liver. Med. Phys..

[pmbae222cbib63] Rosnitskiy P B, Khokhlova T D, Schade G R, Sapozhnikov O A, Khokhlova V A (2024). Treatment planning and aberration correction algorithm for HIFU ablation of renal tumors. IEEE Trans. Ultrason. Ferroelectr. Freq. Control.

[pmbae222cbib64] Rosnitskiy P B, Vysokanov B A, Gavrilov L R, Sapozhnikov O A, Khokhlova V A (2018). Method for designing multielement fully populated random phased arrays for ultrasound surgery applications. IEEE Trans. Ultrason. Ferroelectr. Freq. Control.

[pmbae222cbib65] Rosnitskiy P, Thomas G, Lee G, Khokhlova V, Sapozhnikov O, Schade G, Morrison K, Chavez F, Khokhlova T (2025). A fully populated transrectal array for boiling histotripsy ablation of the prostate. IEEE Trans. Ultrason. Ferroelectr. Freq. Control.

[pmbae222cbib66] Roth H R, Lu L, Seff A, Cherry K M, Hoffman J, Wang S, Liu J, Turkbey E, Summers R M, Golland P, Hata N, Barillot C, Hornegger J, Howe R (2014). A new 2.5D representation for lymph node detection using random sets of deep convolutional neural network observations. Medical Image Computing and Computer-Assisted Intervention—MICCAI 2014.

[pmbae222cbib67] Sandilos G, Butchy M V, Koneru M, Gongalla S, Sensenig R, Hong Y K (2024). Histotripsy—hype or hope? review of innovation and future implications. J. Gastrointest. Surg..

[pmbae222cbib68] Seff A, Lu L, Barbu A, Roth H, Shin H-C, Summers R M, Navab N, Hornegger J, Wells W M, Frangi A (2015). Leveraging mid-level semantic boundary cues for automated lymph node detection. Medical Image Computing and Computer-Assisted Intervention—MICCAI 2015.

[pmbae222cbib69] Seff A, Lu L, Cherry K M, Roth H R, Liu J, Wang S, Hoffman J, Turkbey E B, Summers R M, Golland P, Hata N, Barillot C, Hornegger J, Howe R (2014). 2D view aggregation for lymph node detection using a shallow hierarchy of linear classifiers. Medical Image Computing and Computer-Assisted Intervention—MICCAI 2014.

[pmbae222cbib70] Smolock A R, White S B, Rilling W S, Ziemlewicz T J, Laeseke P F, Vlaisavljevich E, Xu Z, Lee F T (2022). The development of histotripsy for the treatment of liver tumors. Adv. Clin. Radiol..

[pmbae222cbib71] Stocker G E, Lundt J E, Sukovich J R, Miller R M, Duryea A P, Hall T L, Xu Z (2022). A modular, kerf-minimizing approach for therapeutic ultrasound phased array construction. IEEE Trans. Ultrason. Ferroelectr. Freq. Control.

[pmbae222cbib72] Thomas G P L, Khokhlova T D, Bawiec C R, Peek A T, Sapozhnikov O A, O’Donnell M, Khokhlova V A (2021). Phase-aberration correction for HIFU therapy using a multielement array and backscattering of nonlinear pulses. IEEE Trans. Ultrason. Ferroelectr. Freq. Control.

[pmbae222cbib73] Thomas G P L, Khokhlova T D, Sapozhnikov O A, Wang Y-N, Totten S, Khokhlova V A (2022). *In vivo* aberration correction for transcutaneous HIFU therapy using a multi-element array. IEEE Trans. Ultrason. Ferroelectr. Freq. Control.

[pmbae222cbib74] Treeby B E, Cox B T (2010). k-Wave: MATLAB toolbox for the simulation and reconstruction of photoacoustic wave fields. J. Biomed. Opt..

[pmbae222cbib75] Treeby B E, Cox B T (2010). Modeling power law absorption and dispersion for acoustic propagation using the fractional Laplacian. J. Acoust. Soc. Am..

[pmbae222cbib76] Treeby B E, Jaros J, Rendell A P, Cox B T (2012). Modeling nonlinear ultrasound propagation in heterogeneous media with power law absorption using a *k* -space pseudospectral method. J. Acoust. Soc. Am..

[pmbae222cbib77] Tsang S H, Ma K W, She W H, Chu F, Lau V, Lam S W, Cheung T T, Lo C M (2021). High-intensity focused ultrasound ablation of liver tumors in difficult locations. Int. J. Hyperth..

[pmbae222cbib78] Tsysar S A, Rosnitskiy P B, Asfandiyarov S A, Petrosyan S A, Khokhlova V A, Sapozhnikov O A (2024). Phase correction of the channels of a fully populated randomized multielement therapeutic array using the acoustic holography method. Acoust. Phys..

[pmbae222cbib79] Vidal-Jové J, Serres-Créixams X, Ziemlewicz T J, Cannata J M (2021). Liver histotripsy mediated abscopal effect—case report. IEEE Trans. Ultrason. Ferroelectr. Freq. Control.

[pmbae222cbib80] Vlaisavljevich E, Gerhardson T, Hall T, Xu Z (2017). Effects of f-number on the histotripsy intrinsic threshold and cavitation bubble cloud behavior. Phys. Med. Biol..

[pmbae222cbib81] Vlaisavljevich E, Kim Y, Owens G, Roberts W, Cain C, Xu Z (2014). Effects of tissue mechanical properties on susceptibility to histotripsy-induced tissue damage. Phys. Med. Biol..

[pmbae222cbib82] Vlaisavljevich E, Xu Z, Arvidson A, Jin L, Roberts W, Cain C (2015). Effects of thermal preconditioning on tissue susceptibility to histotripsy. Ultrasound Med. Biol..

[pmbae222cbib83] Wagner M G (2023). An x-ray C-arm guided automatic targeting system for histotripsy. IEEE Trans. Biomed. Eng..

[pmbae222cbib84] Wah T M (2023). A multi-centre, single arm, non-randomized, prospective European trial to evaluate the safety and efficacy of the Histosonics system in the treatment of primary and metastatic liver cancers (#HOPE4LIVER). CardioVascular Interventional Radiol..

[pmbae222cbib85] Wehrle C J (2025). The first international experience with histotripsy: a safety analysis of 230 cases. J. Gastrointest. Surg..

[pmbae222cbib86] White P J, Andre B, McDannold N, Clement G T (2008). A pre-treatment planning strategy for high-intensity focused ultrasound (HIFU) treatments.

[pmbae222cbib87] Wise E S, Cox B T, Jaros J, Treeby B E (2019). Representing arbitrary acoustic source and sensor distributions in Fourier collocation methods. J. Acoust. Soc. Am..

[pmbae222cbib88] Wu F, Thomas J, Fink M (1992). Time reversal of ultrasonic fields. Il. Experimental results. IEEE Trans. Ultrason. Ferroelectr. Freq. Control.

[pmbae222cbib89] Xu Z, Hall T L, Vlaisavljevich E, Lee F T (2021). Histotripsy: the first noninvasive, non-ionizing, non-thermal ablation technique based on ultrasound. Int. J. Hyperth..

[pmbae222cbib90] Xu Z, Raghavan M, Hall T L, Mycek M-A, Fowlkes J B, Cain C A (2008). Evolution of bubble clouds induced by pulsed cavitational ultrasound therapy—histotripsy. IEEE Trans. Ultrason. Ferroelectr. Freq. Control.

[pmbae222cbib91] Yeats E M (n.d.). k-Wave Simulation Code and Data for Histotripsy Liver Treatment Envelope Study. University of Michigan - Deep Blue Data.

[pmbae222cbib92] Yeats E, Gupta D, Xu Z, Hall T L (2022). Effects of phase aberration on transabdominal focusing for a large aperture, low f-number histotripsy transducer. Phys. Med. Biol..

[pmbae222cbib93] Yeats E, Hall T L (2023). Aberration correction in abdominal histotripsy. Int. J. Hyperth..

[pmbae222cbib94] Yeats E, Lu N, Stocker G, Komaiha M, Sukovich J R, Xu Z, Hall T L (2024). *In vivo* cavitation-based aberration correction of histotripsy in porcine liver. IEEE Trans. Ultrason. Ferroelectr. Freq. Control.

[pmbae222cbib95] Yeats E, Lu N, Sukovich J R, Xu Z, Hall T L (2023). Soft tissue aberration correction for histotripsy using acoustic emissions from cavitation cloud nucleation and collapse. Ultrasound Med. Biol..

[pmbae222cbib96] Yuldashev P V, Shmeleva S M, Ilyin S A, Sapozhnikov O A, Gavrilov L R, Khokhlova V A (2013). The role of acoustic nonlinearity in tissue heating behind a rib cage using a high-intensity focused ultrasound phased array. Phys. Med. Biol..

[pmbae222cbib97] Ziemlewicz T J (2025). The #HOPE4LIVER single-arm pivotal trial for histotripsy of primary and metastatic liver tumors: 1-year update of clinical outcomes. Ann. Surg..

[pmbae222cbib98] Zubair M, Dickinson R J (2021). 3D synthetic aperture imaging with a therapeutic spherical random phased array for transcostal applications. Phys. Med. Biol..

